# Serogroup B Meningococcal Sepsis and Meningitis Associated With Meningococcal Acute Otitis Media, and Paranasal Sinusitis in an Infant: A Case Report

**DOI:** 10.7759/cureus.38430

**Published:** 2023-05-02

**Authors:** Enrique Chacon-Cruz, Erika Z Lopatynsky

**Affiliations:** 1 Pediatric Infectious Diseases, Hospital General de Tijuana, Tijuana, MEX; 2 Vaccines and Infectious Diseases, Think Vaccines LLC, Houston, USA; 3 Family Medicine and Public Health, University of California San Diego, San Diego, USA

**Keywords:** meningococcal vaccine, serogroup b neisseria meningitidis, meningococcal paranasal sinusitis, meningococcal acute otitis media, meningococcal meningitis

## Abstract

Meningococcal disease (MD) is a potentially lethal condition. Typically, following infection, MD manifests with high fever, with signs and symptoms of severe septicemia with or without purpura, and in more than half of cases with meningitis. Acute otitis media (AOM) caused by *Neisseria meningitidis* has scarcely been reported, mostly without severe MD, and there are no reports of meningococcal paranasal sinusitis (PS). We present the case of a previously healthy 11-month-old infant who started with fever and cough and further developed intense irritability and right spontaneous purulent otorrhea, with subsequent increased fever and seizures. Blood, cerebrospinal, and middle ear fluid cultures were positive for *N. meningitidis* serogroup B, and a CT scan showed both maxillary and ethmoidal sinusitis. Intravenous ceftriaxone was administered for eight days, and three months following discharge, no sequelae were identified. This is the first report of a patient with MD associated with sepsis, meningitis, AOM, and PS.

## Introduction

Meningococcal disease (MD) results from a contagious infection by *Neisseria meningitidis* transmitted from human to human by respiratory droplets or nasopharynx secretions [1,2]. Pathogenesis of MD is directly related to the presence of a capsule, therefore evading opsonophagocytosis, allowing the bacteria to invade the bloodstream (septicemia), including the central nervous system (meningitis) [1,2]. MD is usually accountable for a broad, very aggressive clinical spectrum, and usually, signs and symptoms occur within 1-14 days following infection, albeit often appear in the first seven days. Early symptoms can be similar to other bacterial infections, making prompt recognition frequently problematic. Fever, nausea, vomiting, abrupt headache, photophobia, neck stiffness, and altered mental state are typical symptoms, with young infants manifesting more unspecified symptoms such as intense irritability, food refusal, and hypothermia, among others. At least half of the patients develop acute purpura fulminans, characterized by purpuric skin lesions and petechia, and may rapidly lead to adrenal glands hemorrhage (Waterhouse-Fredericksen syndrome), shock, and death [1-3].

Less common symptoms are related to pneumonia, conjunctivitis, acute otitis media (AOM), epiglottitis, urethritis, arthritis, and pericarditis [1-3]. Albeit all of the latter usually manifest during the severe acute presentation of MD, these less serious clinical presentations can also be manifested before septicemic/meningeal MD or even as isolated manifestations [1-3].

Although meningococcal AOM with and without meningitis has been reported a few times [4-6], to date, this is the first documented case of MD with confirmed bacteremia, meningitis, AOM, and paranasal sinusitis (PS), in which *N. meningitidis* serogroup B was isolated from blood, cerebrospinal fluid (CSF), and middle ear fluid.

## Case presentation

An 11-month-old female was admitted on October 12, 2017, with a three-day history of non-quantified fever, cough, rhinorrhea, vomiting, and on the following day, one episode of generalized seizures at home that lasted presumably less than one minute. Physical findings at admission showed a heart rate of 134 per minute, blood pressure of 80/40 mmHg, oral temperature of 39˚Celsius, and breath rate of 36 per minute. The patient was subconscious and partially responsive to tactile stimuli. Her pupils were normal, and she had very marked neck stiffness. Purpura, petechial lesions, and signs of hypoperfusion were not found.

In addition, purulent right otorrhea was seen at admission. The right ear was not possible to examine due to a profuse purulent otorrhea. The left ear, as well as nose and throat, did not show any clinical abnormalities; we re-interrogated the patient’s mother, who said this event had occurred between three to four hours before seizures presented. Further clinical (prior hospitalizations, recurrent infections, etc.) and family history (immunodeficiencies, stillbirths, etc.) were denied. In fact, the patient was an only child; her father worked as an accountant, and her mother was a housewife. Before admission, only paracetamol was given to the infant to control the fever.

The baby had all vaccines according to the Mexican National Immunization Program (NIP) for her age (which included two doses of the 13-valent pneumococcal conjugate vaccine); however, she had never received any meningococcal vaccine since these immunogens are not yet part of the Mexican NIP.

Laboratory results (Table 1) at the Emergency Room showed marked leukocytosis with granulocytosis, an elevated procalcitonin, normal serum electrolytes, HIV negative (by enzyme-linked immunosorbent assay (ELISA)), and a CSF showing more than 10,000 leukocytes with 70% of granulocytes, 212 mg/dL of proteins, and zero glucose, consistent with bacterial meningitis. 

**Table 1:** Patient´s laboratory findings on admission ALT: alanine aminotransferase; AST: aspartate aminotransferase; BUN: blood urea nitrogen; HIV: human immunodeficiency virus; ELISA: enzyme-linked immunosorbent assay

CELL BLOOD COUNT	RESULT	INTERPRETATION
Leukocytes	25,600/μL	Elevated
Granulocytes	19,400/μL	Elevated
Lymphocytes	4,800/μL	Normal
Eosinophils	1,100/μL	Normal
Basophils	300/μL	Normal
Platelets	332,000/μL	Normal
Hemoglobin	15 g/dl	Normal
Erythrocyte Sedimentation Rate	48 mm/hr	Elevated
SERUM ELECTROLYTES		
Sodium	144 mEq/L	Normal
Potassium	5.1 mEq/L	Normal
Chloride	102 mEq/L	Normal
Calcium	9.8 mg/dl	Normal
Magnesium	2.1 mg/dl	Normal
OTHER SERUM TESTS		
ALT	38 U/L	Normal
AST	41 U/L	Normal
BUN	11 mg/dl	Normal
Creatinine	0.8 mg/dl	Normal
Glucose	98 mg/dl	Normal
Albumin	4.1 g/L	Normal
Procalcitonin	4.5 μg/L	Very elevated
HIV by ELISA	Negative	Normal
CEREBROSPINAL FLUID		
Leukocytes	10,680/μL	Very elevated
Granulocytes	7,476/μL	Very elevated
Proteins	212.8 mg/dL	Elevated
Glucose	0.0 mg/dl	Very low (zero)
Gram stain	Gram-negative diplococci	Presence of bacterial

**Figure 1:**
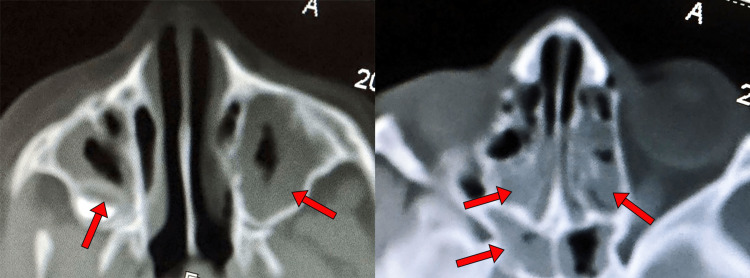
Ethmoidal and maxillary sinusitis by CT scan

Immediate administration of intravenous 60 mg/kg of vancomycin and 100 mg/kg ceftriaxone was started (to treat *Streptococcus pneumoniae* and *N. meningitidis* empirically), along with all intensive care unit protocols of a child with sepsis and meningitis. A computerized tomography (CT) scan showed no brain lesions; however, clear tomographic signs of maxillary and ethmoidal sinusitis were found (Figure 1).

**Figure 2:**
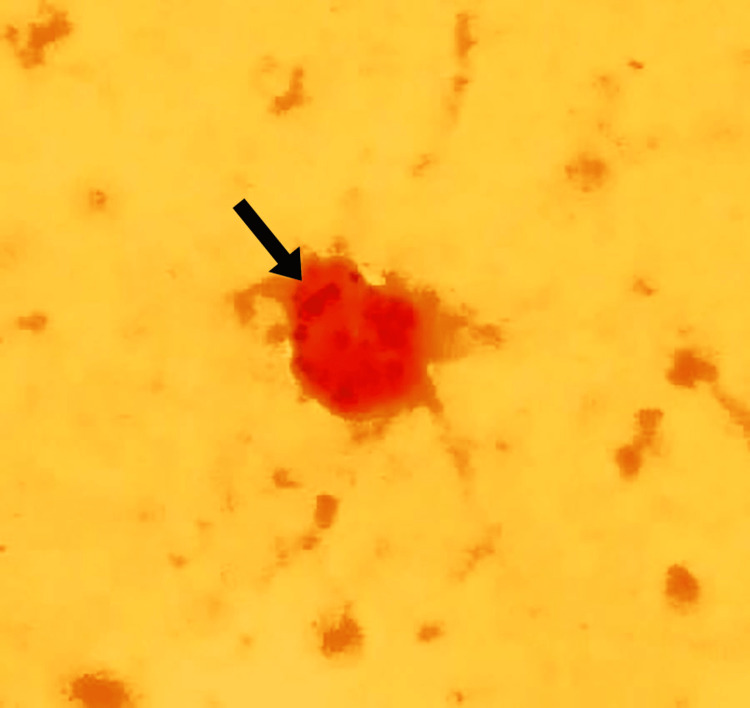
Gram stain from CSF; the arrow indicates intracellular Gram-negative diplococci CSF: cerebrospinal fluid

A Gram stain from CSF showed intracellular Gram-negative diplococci (Figure 2), and, on the next day following admission, cultures from blood, CSF, and middle ear fluid were positive for *N. meningitidis* serogroup B, susceptible to penicillin and third generation cephalosporins. No cultures were obtained from either maxillary or ethmoidal sinuses since the technique was invasive and irrelevant to the patient´s potential outcome. Vancomycin was then suspended, and intravenous ceftriaxone continued. Two days of oral rifampin were then administered to both parents, as indicated [1-3]. 

After two days of treatment, the fever disappeared, the patient became conscious, and all other signs and symptoms resolved. She was discharged eight days following admission.

Physical, electroencephalographic, and neurological examinations were performed after three months of discharge, with no apparent sequelae (including normal electroencephalogram) and the right ear drum without signs of perforation following otoscopy. Serum immunoglobulins and complement were performed with normal values. A new CT scan for the brain and paranasal sinuses was planned, but the patient never returned for a follow-up.

## Discussion

Conducting enhanced active surveillance at the General Hospital of Tijuana, Mexico, for 13 years [7] enabled us to identify all MD cases, including those with an uncommon presentation like this case and others [8]. Respiratory infections due to *N. meningitidis* have been well documented in the literature [1-6,9], from which pneumonitis with or without pleural effusion is by far the better described by Feldman and Anderson [9].

Primary meningococcal pneumonia occurs in 5-10% of patients with meningococcal infection and is indistinguishable clinically from pneumonia caused by other common pathogens. Fever, chills, and pleuritic chest pain are the most common symptoms, occurring in > 50% of cases [9]. As with *S. pneumoniae*, meningococcus colonizes the nasopharynx; however, given the seemingly lesser virulence of the meningococcus relative to that of exotoxin-producing bacterial respiratory pathogens such as *S. pneumoniae*, *Staphylococcus aureus,* and *Pseudomonas aeruginosa*, meningococcus may require a trigger to achieve full pathogenicity, which could be an associated virus with/without compromised immunity, or a favorable climatic condition for *N. meningitidis *such as the hot and dry seasonable Santa Ana winds, as we have previously published [10].

The association of MD and AOM was initially described in 1985 by Hatch et al. in New Zealand; however, information on all cases is scarce, and most are not associated with systemic MD [4]. Edwards et al., also in 1985, described eight mild cases of meningococcal infection, five with AOM. Interestingly, all but two patients were treated as outpatients, one even with meningitis cured without inpatient management, and none developed signs of sepsis or purpura [5]. Furthermore, in a study done by Poetker et al., in the USA, from 292 patients undergoing tympanostomy and middle ear cultures, less than 11% of middle fluid samples were positive for *N. meningitidis*; however, all patients had no symptoms consistent with MD [6].

This is the first confirmed MD case associated with sepsis, meningitis, purulent AOM, and PS. Though admitted severely ill, the patient responded very rapidly to medical interventions and with apparent no sequelae following three months of hospital discharge. Indeed, the question of whether an upper respiratory infection in MD could be a factor of good prognosis remains unclear and, to date, very difficult to address. 

Endemicity in Tijuana, Mexico, has been reported in previous studies [7,11,12], including the appearance of an outbreak [13]. The economic burden of it is substantial [14]. All of the latter urges for universal meningococcal vaccination in the region.

## Conclusions

This is the first case of confirmed serogroup B systemic MD associated with meningococcal meningitis, purulent AOM, and PS, a very unusual presentation of MD. By implementing active surveillance at the General Hospital of Tijuana, Mexico, for 13 years, proper identification of all MD cases has significantly improved, including recognition of patients with common and uncommon presentations, like this case.

In addition, the Tijuana Baja-California region in Mexico has proved to be endemic to MD and implementation of universal meningococcal vaccination is important in the region. Such rare MD cases can be more easily identified when proper active surveillance is performed, as in our hospital, and this should be encouraged. This report would hopefully positively affect the identification of such cases and contribute to promoting preventive measures such as universal meningococcal vaccination. 
